# Inhibition of Endoplasmic Reticulum Stress Improves Acetylcholine-Mediated Relaxation in the Aorta of Type-2 Diabetic Rats

**DOI:** 10.3390/molecules27165107

**Published:** 2022-08-11

**Authors:** Sagir Mustapha, Ahmad Khusairi Azemi, Wan Amir Nizam Wan Ahmad, Aida Hanum Ghulam Rasool, Mohd Rais Mustafa, Siti Safiah Mokhtar

**Affiliations:** 1 Department of Pharmacology, School of Medical Sciences, Universiti Sains Malaysia, Kota Bharu 16150, Kelantan, Malaysia; 2 Department of Pharmacology and Therapeutics, Ahmadu Bello University, Zaria 810107, Kaduna, Nigeria; 3 Institute of Marine Biotechnology, Universiti Malaysia Terengganu, Kuala Terengganu 21030, Terengganu, Malaysia; 4 Biomedicine Programme, School of Health Sciences, Universiti Sains Malaysia, Kota Bharu 16150, Kelantan, Malaysia; 5 Department of Pharmacology, Faculty of Medicine, University of Malaya, Kuala Lumpur 50603, Selangor, Malaysia

**Keywords:** endothelial function, ER stress, tauro-ursodeoxycholic acid (TUDCA), type-2 diabetes, vasculopathy

## Abstract

Endoplasmic reticulum (ER) stress contributes to insulin resistance and macro- and microvascular complications associated with diabetes. This study aimed to evaluate the effect of ER stress inhibition on endothelial function in the aorta of type-2 diabetic rats. Type-2 diabetes was developed in male Sprague–Dawley rats using a high-fat diet and low-dose streptozotocin. Rat aortic tissues were harvested to study endothelial-dependent relaxation. The mechanisms for acetylcholine-mediated relaxation were investigated using pharmacological blockers, Western blotting, oxidative stress, and inflammatory markers. Acetylcholine-mediated relaxation was diminished in the aorta of diabetic rats compared to control rats; supplementation with TUDCA improved relaxation. In the aortas of control and diabetic rats receiving TUDCA, the relaxation was mediated via eNOS/PI3K/Akt, NAD(P)H, and the K_ATP_ channel. In diabetic rats, acetylcholine-mediated relaxation involved eNOS/PI3K/Akt and NAD(P)H, but not the K_ATP_ channel. The expression of ER stress markers was upregulated in the aorta of diabetic rats and reduced with TUDCA supplementation. The expression of eNOS and Akt were lower in diabetic rats but were upregulated after supplementation with TUDCA. The levels of MDA, IL-6, and SOD activity were higher in the aorta of the diabetic rats compared to control rats. This study demonstrated that endothelial function was impaired in diabetes, however, supplementation with TUDCA improved the function via eNOS/Akt/PI3K, NAD(P)H, and the K_ATP_ channel. The improvement of endothelial function was associated with increased expressions of eNOS and Akt. Thus, ER stress plays a crucial role in the impairment of endothelial-dependent relaxation. Mitigating ER stress could be a potential strategy for improving endothelial dysfunction in type-2 diabetes.

## 1. Introduction

Type-2 diabetes is a chronic metabolic disease caused by the combination of hereditary and environmental factors. The prevalence of type-2 diabetes has reached epidemic proportions across the globe, affecting the low- and middle-income classes of developed countries. Diabetes poses a significant public health risk because it causes severe micro- and macrovascular complications in those affected [1]. A hallmark of these vascular complications is the development of endothelial dysfunction, characterized by impaired endothelium-dependent relaxation on both conduit and resistance arteries in human and animal models of type-2 diabetes. A previous study summarized the possibility of involvement of endoplasmic reticulum (ER) stress in diabetic endothelial dysfunction and its associated vascular complications [2].

The endothelium, a monolayer of cells lining the intima of blood vessels, regulates vascular tone by releasing endothelium-derived relaxing factors (EDRF) and contracting factors (EDCF). Nitric oxide (NO) is the major and potent EDRF that modulates endothelium-dependent relaxation in most vessels [3]. Acetylcholine, shear pressure, and bradykinin lead to the formation of the calcium–calmodulin complex (CaM) that binds to endothelial NO synthase (eNOS). The interaction between CaM and eNOS allows phosphorylated protein kinase B (Akt) to attach to eNOS. This association leads to the oxidation of amino acid L-arginine to generate NO and L-citrulline [4]. In addition, NAD(P)H oxidase and the K_ATP_ channel in the endothelium contribute to endothelium-dependent relaxation or vascular tone. The endothelial NAD(P)H oxidase physiologically produces reactive oxygen species (ROS) [superoxide anion (O_2_^−^) and hydrogen peroxide (H_2_O_2_)] as a primary function rather than a consequence. ROS produced by NADPH oxidase is not the byproduct of other biological reactions [5]. Similarly, ER generates H_2_O_2_ as the main ROS during protein folding in the ER lumen [6]. NAD(P)H oxidase (NOX4) is a well-known ER resident that generates H_2_O_2_ and O_2_ [5]. Collectively, NAD(P)H oxidase and ER generate more ROS in diabetes conditions. The endothelium K_ATP_ channels play a role in various physiological functions, such as regulating vascular tone and blood flow [7]. Similarly, the K_ATP_ channels consist of four regulatory sulphonylurea receptor subunits [8]. H_2_O_2_ is an essential signaling molecule that stimulates the opening of K_ATP_ channels [9]. Activation of the endothelial K_ATP_ channel enhances the entry of Ca^2+^ into endothelial cells [10]. This leads to an increase in endothelial cell Ca^2+^, which results in the formation of CaM [11]. The formation of CaM can lead to the activation of eNOS in calcium-dependent pathways. Endothelial dysfunction occurs due to a reduction in eNOS activity and/or expression, and decreased NO bioavailability resulting in part from enhanced oxidative stress [12].

The ER is a cell organelle that is the site for protein synthesis, translocation, biosynthesis, folding, post-translational, signaling podium, and molecular chaperone folding mechanisms [13,14]. The perturbation of ER homeostasis leads to misfolding or unfolding proteins, causing ER stress. In response to ER stress, the cell activates a signaling network called unfolded protein response (UPR), which attempts to restore normal ER function. The three main signaling networks are inositol-requiring kinase 1 (IRE1), protein kinase-like ER kinase (PERK), and activating transcription factor 6 (ATF6), which are ER stress sensors. When adaptations to restore homeostasis fails, the UPR effectors can trigger a cascade of inflammatory responses and increase reactive oxygen species generation, which finally leads to insulin dysfunction and cell death via the activation of many pro-apoptotic signals [15,16]. Chronic UPR activation has emerged over the last decade as an important contributor to metabolic diseases; compelling evidence shows that ER stress is involved in the pathogenesis of endothelial dysfunction. UPR activation may impair endothelial function directly, either by altering the synthesis and molecular signalling of vasoactive substances, such as NO, or indirectly by inducing other signalling networks, such as inflammation and oxidative stress [17]. A previous study highlighted the role of ER stress and implicated proteins involved in diabetes-related pathogenesis, including PERK, IRE1, and ATF6 [18].

It has been demonstrated that treatment with the ER stress inducer, tunicamycin, leads to impairment of endothelium-dependent relaxation in mice aortas and mesenteric resistance arteries [19]. Similarly, in the aorta of young female rats, tunicamycin-induced ER stress has been reported to be linked to impaired vasomotor responsiveness to insulin and endothelial dysfunction [20]. Choi et al. in 2016 [21,22] reported increases in ER stress at the coronary arteries of type-2 diabetes and hypertensive mice. Inhibition of ER stress with tauro-ursodeoxycholic acid (TUDCA) were associated with the normalization of the myogenic response and endothelium-mediated relaxation in these mice. Similarly, Spitler et al. in 2013 [23] found that increased ER stress was present in the aorta of spontaneously hypertensive rats (SHR); suppression of ER stress reduced endothelium-dependent contraction in the aorta of these SHR. Kassan et al. in 2013 [19] also reported on the reduction of cardiac damage and improvement of vascular function in the aorta of hypertensive mice after inhibition of ER stress using TUDCA and 4-phenylbutyric acid (PBA).

To date, scarce data are available on the significance and the role of ER stress in modulating various molecular mechanisms underlying endothelial dysfunction in type-2 diabetes. Thus, the aim of this study is to determine the involvement of ER stress on molecular mechanisms of NO-mediated endothelial-dependent relaxation in the aorta of rats with type-2 diabetes.

## 2. Results

### 2.1. Effect of ER Stress Inhibition on Blood Pressure, Body Weight, Blood Glucose Level, and Food Consumption

**Figure 1:**
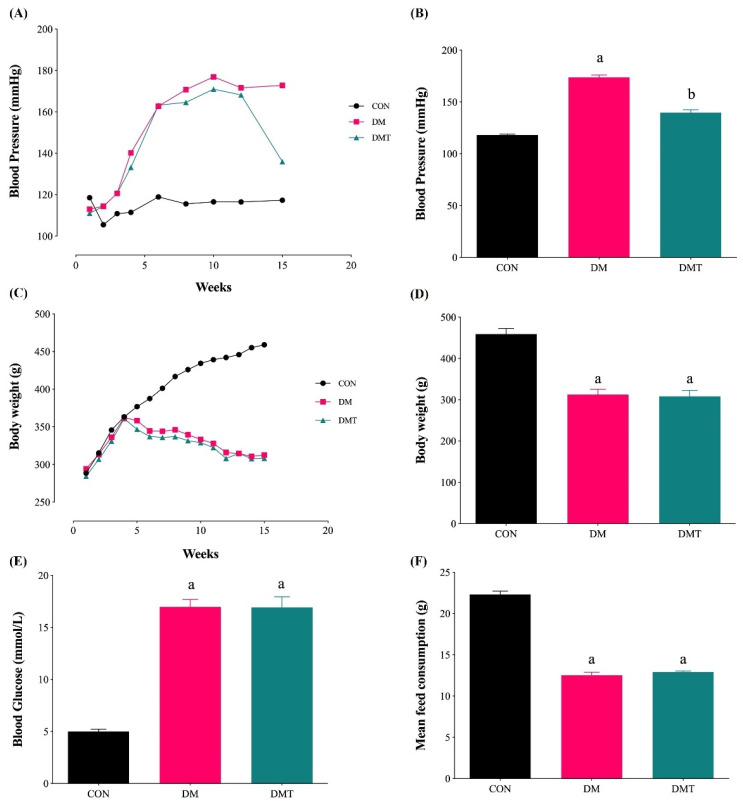
BP, body weight, blood glucose level, and mean food consumption in experimental groups. (**A**) BP changes throughout time, (**B**) BP at week 15, (**C**) body weight changes throughout time, (**D**) body weight at week 15, (**E**) blood glucose level at week 15, and (**F**) mean food consumption for the whole study duration (n = 9). Abbreviations: CON; Control, DM; Diabetes, DMT; Diabetic receiving TUDCA, and TUDCA; Tauro-ursodeoxycholic acid. ^a^ *p* ˂ 0.05, vs. CON, ^b^ *p* ˂ 0.05, vs. DM.

The blood pressure (BP) and body weight were significantly higher and lower in the DM group (173.77 ± 2.38 mmHg, and 312.56 ± 13.06 g, respectively) compared to the CON group (118.18 ± 0.75 mmHg, and 459.00 ± 13.77 g, respectively) (Figure 1A–D). The blood glucose level in the DM group was significantly higher, and the mean food consumption was lower (16.98 ± 0.73 mmol/L and 12.53 ± 0.36 g, respectively) compared to the CON groups (5.01 ± 0.22 mmol/L and 22.31 ± 0.42 g, respectively) (Figure 1E,F). Inhibition of ER stress using TUDCA (150 mg/kg/day) in diabetic rats did not affect the blood glucose level, mean food consumption, and body weight, but was able to reduce the BP (16.93 ± 1.02 mmol/L, 12.91 ± 0.14 g, 308.33 ± 14.21 g and 139.82 ± 2.80 mmHg, respectively).

### 2.2. Effect of ER Stress Inhibition on Endothelium-Dependent Relaxation

**Figure 2:**
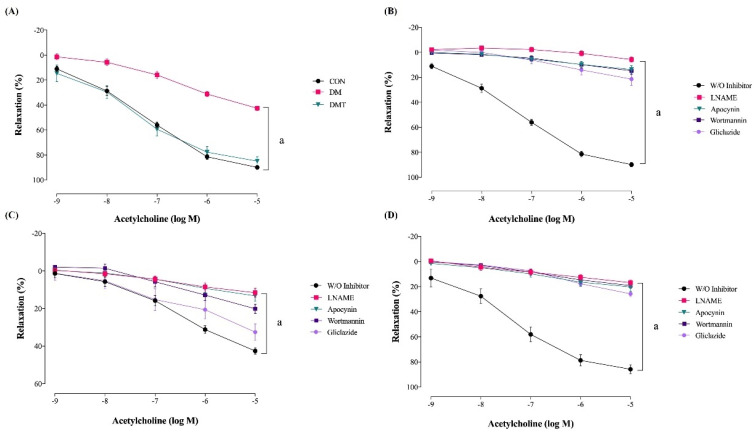
(**A**) Concentration response curves to acetylcholine in the aorta of the experimental rats. Concentration response curves to acetylcholine in the presence of pharmacological inhibitors in the aorta of (**B**) CON rats, (**C**) DM rats, and (**D**) DMT rats (n = 9). Relaxations are expressed as the percentage of the contraction induced by phenylephrine. Abbreviation: CON; Control, DM; Diabetes, DMT; Diabetes receiving TUDCA, LNAME; L-Nitro-Arginine Methyl Ester, and W/O; without inhibitor.

The maximal relaxation due to acetylcholine in the aorta of DM was significantly impaired compared to CON rats (Table 1, Figure 2A). TUDCA supplementation to diabetic rats (DMT group) significantly improved maximal relaxation compared to DM rats. In the aorta of CON rats, maximal relaxation was almost abolished in the presence of pharmacological inhibitors like LNAME, wortmannin, apocynin, and gliclazide (Table 1, Figure 2B). In the aorta of DM rats, the maximal relaxation was reduced in the presence of LNAME, wortmannin, and apocynin. However, there was no significant difference in the relaxation after incubation with gliclazide (Table 1, Figure 2C). The maximal relaxation of the aorta in DMT rats was almost abolished in the presence of LNAME, wortmannin, apocynin, and gliclazide, which is similar to the responses in CON rats (Table 1, Figure 2D).

### 2.3. Effect of ER Stress Inhibition on the Protein Expression of BiP, PERK, eNOS, and Akt

**Figure 3:**
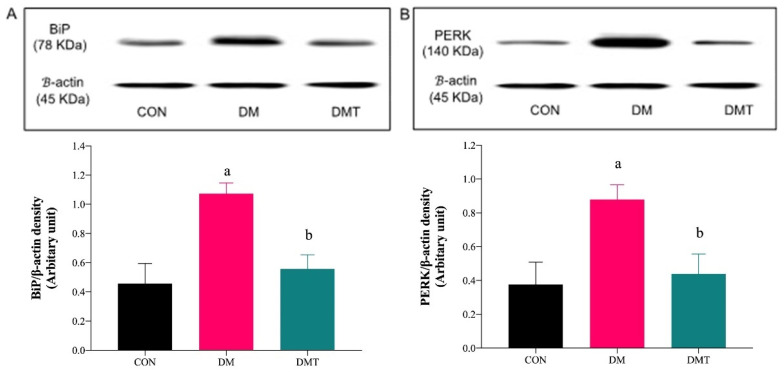
Representative blots showing the density of the protein bands for (**A**) BiP and (**B**) PERK in rat aorta. Graphical representations of the data are normalised to β-actin (n = 9). ^a^ *p* < 0.05 vs. CON, ^b^ *p* < 0.05 vs. DM. Abbreviation: CON; Control, DM; Diabetes, DMT; Diabetes receiving TUDCA, BiP; Binding immunoglobulin protein, and PERK; Protein kinase-like ER kinase.

**Figure 4:**
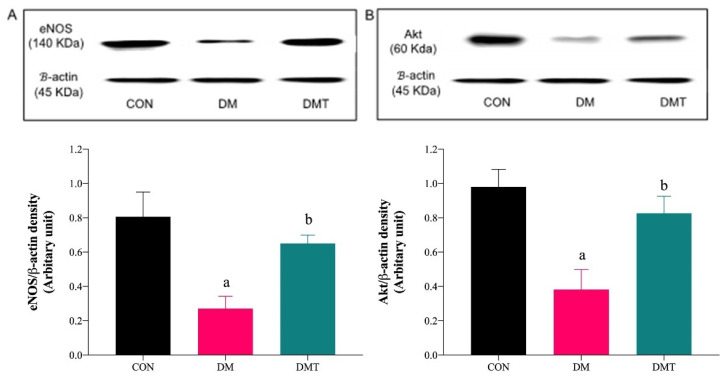
Representative blots showing the density of the protein bands for (**A**) eNOS and (**B**) Akt in rat aorta. Graphical representations of the data are normalised to β-actin (n = 9). ^a^ *p* < 0.05 vs. CON, ^b^ *p* < 0.05 vs. DM. Abbreviation: CON; Control, DM; Diabetes, DMT; Diabetes receiving TUDCA, eNOS; Endothelial nitric oxide synthase, and Akt; Protein Kinase B.

BiP and PERK protein expressions were upregulated in DM compared to CON rats. The expression of these two markers were lowered after supplementation with TUDCA (Figure 3A,B). Expression of Akt and eNOS proteins were lowered in DM compared to CON rats, and the expressions were increased after supplementation with TUDCA (Figure 4A,B).

### 2.4. Effect of ER Stress Inhibition on SOD Activity and MDA Level

**Figure 5:**
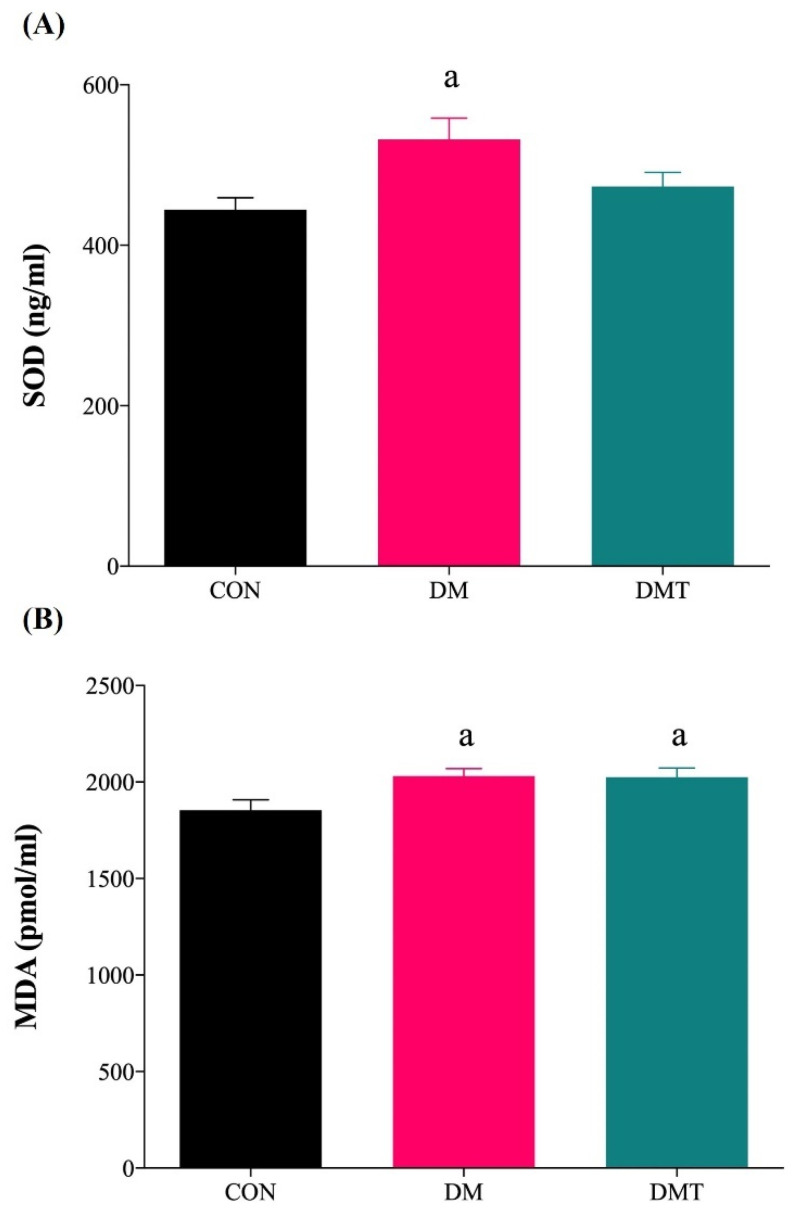
(**A**) SOD activity and (**B**) MDA level in the aorta of experimental rats (n = 9). ^a^ *p* ˂ 0.05 vs. CON. Abbreviation: CON; Control, DM; Diabetes, DMT; Diabetes receiving TUDCA, SOD; Superoxide dismutase, and MDA; Malondialdehyde.

The SOD activity and MDA levels were significantly higher in the aorta of DM compared to CON rats. TUDCA supplementation in diabetic rats reduced the SOD activity (DMT: 453.73 ± 15.16 vs. DM: 531.79 ± 26.75 ng/mL protein) and MDA levels (DMT: 2000.00 ± 45.94 vs. DM: 2046.43 ± 41.02 pmol/mL protein) compared with DM rats, however they were not statistically significant and did not produce full recovery when compared to CON rats (SOD: 444.41 ± 14.90 ng/mL protein and MDA: 1854.76 ± 53.82 pmol/mL protein) (Figure 5A,B).

### 2.5. Effect of ER Stress Inhibition on IL-6 and TNF-α

**Figure 6:**
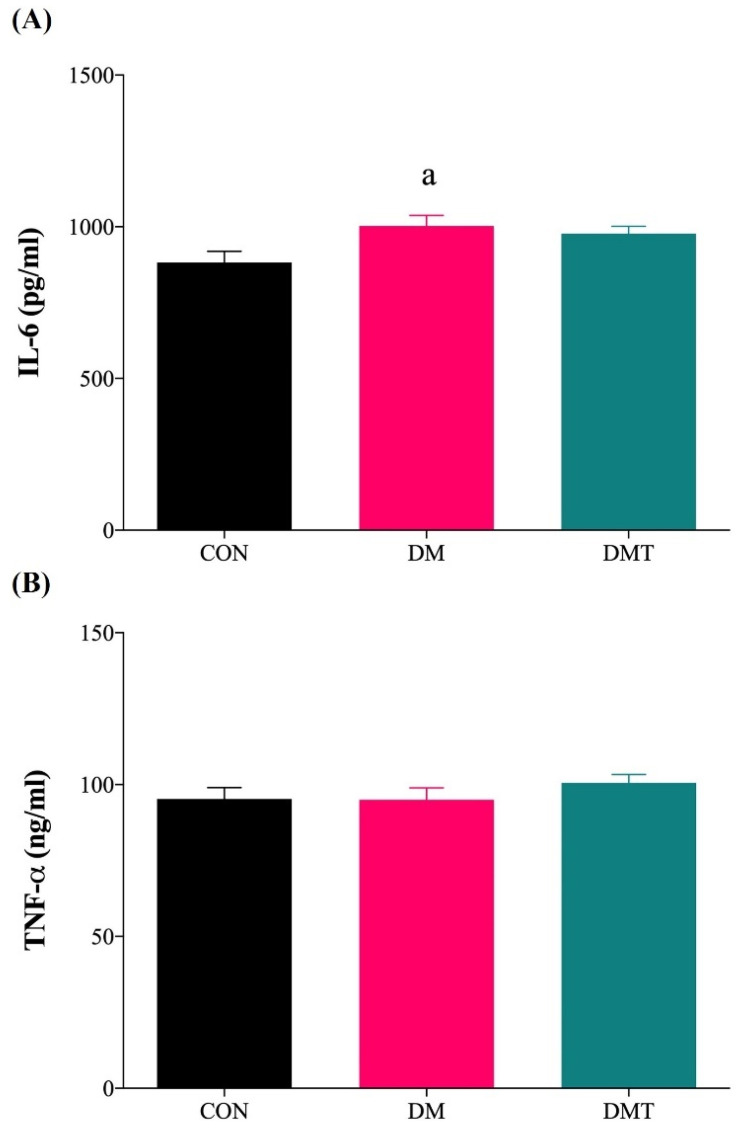
(**A**) IL-6 level and (**B**) TNF-α level in the aorta of experimental rats (n = 9). ^a^ *p* ˂ 0.05 vs. CON. Abbreviation: CON; Control, DM; Diabetes, DMT; Diabetes receiving TUDCA, IL-6; Interleukin-6, and TNF-α; Tumor Necrosis Factor-alpha.

The IL-6 levels were significantly higher in the aorta of DM compared to CON rats (DM: 1003.00 ± 35.52 vs. CON: 882.90 ± 36.02 pg/mL). Administration of TUDCA did not significantly decrease the IL-6 levels in the aorta of DMT compared to DM rats (DMT: 977.80 ± 23.75 vs. DM: 1003.00 ± 35.52 pg/mL) (Figure 6A). The levels of TNF-α were comparable in all experimental groups (DM: 95.02 ± 3.87, CON: 95.26 ± 3.72 and DMT: 100.60 ± 2.74 ng/mL) (Figure 6B).

## 3. Discussion

**Figure 7:**
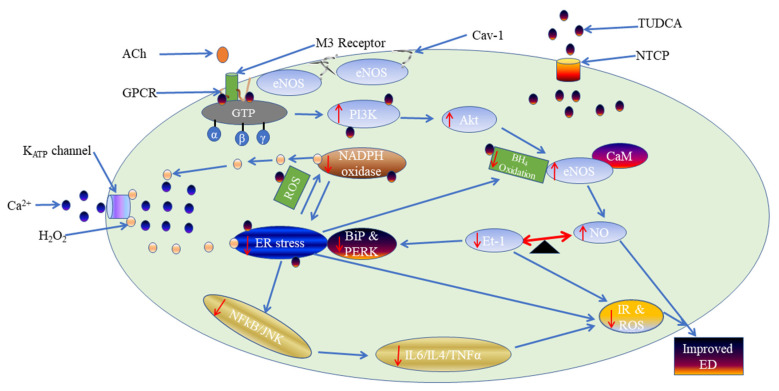
Schematic representation of the interaction between TUDCA, eNOS/PI3K/Akt, NAD(P)H oxidase, and K_ATP_ channel. Abbreviation: ACh; Acetylcholine, M3 receptor; Muscarinic receptor, Cav-1; Caveolin-1, TUDCA; Tauro-ursodeoxycholic acid, NTCP; Sodium/taurocholate co-transporter peptide, K_ATP_ channel; ATP-sensitive potassium channel, Ca^2+^; Calcium ion, H_2_O_2_; Hydrogen peroxide, GPCR; G-protein coupled receptor, GTP; Guanosine triphosphate, eNOS; Endothelial nitric oxide synthase, PI3K; Phosphatidylinositol 3-kinases, Akt; Protein kinase B, CaM; Calcium-calmodulin complex, BH_4_; Tetrahydrobiopterin, NO; Nitric oxide, Et-1; Endothelin-1, IR; Insulin resistance, ROS; Reactive oxygen species, BiP; Binding immunoglobulin protein, PERK; Double-stranded RNA protein kinase-like ER kinase, ER stress; Endoplasmic reticulum stress, NAD(P)H oxidase; Nicotinamide adenine dinucleotide phosphate oxidase, NF-_Κ_B; Nuclear factor-kappa B, JNK; c-Jun N-terminal kinase, IL6; interleukin-6, IL-4; interleukin-4, TNFα; tumour necrosis factor α, and ED; Endothelial dysfunction, Red down arrows indicate decrease, Red upward arrows indicate increase, Blue arrows indicate direction of reaction.

The present study demonstrated that: (a) The protein expression for ER stress markers, BiP and PERK, were upregulated in the aorta of DM compared to control rats, which implies that ER stress was upregulated in DM. Treatment with TUDCA to DM rats reduced ER stress markers (Figure 7); (b) acetylcholine-induced endothelium-dependent relaxation was diminished in the aorta of DM compared to control rats; supplementation with TUDCA to DM rats improved acetylcholine-mediated relaxation (Figure 7); (c) in control rats, acetylcholine-mediated relaxation through eNOS/PI3K/Akt pathway, NAD(P)H oxidase, and the endothelial K_ATP_ channel. In DM rats, acetylcholine-mediated relaxation involved the eNOS/PI3K/Akt pathway and NAD(P)H oxidase, but not the endothelial K_ATP_ channel. Treatment with TUDCA to DM rats showed that acetylcholine-mediated relaxation involved the eNOS/PI3K/Akt pathway, NAD(P)H oxidase, and endothelial K_ATP_ channel, which are similar to those in control rats (Figure 7; (d) the expressions of eNOS and Akt proteins were downregulated in DM compared to control rats and upregulated after treatment with TUDCA; and (e) levels of oxidative stress markers (SOD activity and MDA level) and the pro-inflammatory marker, IL-6, were high in the aorta of DM compared to control rats.

Vascular tone is controlled via the release of various EDRF and EDCF. NO, which is an EDRF, acts as the major contributor to vascular relaxation. Reduced bioavailability of NO leads to impaired endothelium-dependent relaxation. In the present study, the aorta of diabetic rats demonstrated impaired acetylcholine-induced relaxation compared to control rats. This result is consistent with previous studies on the aorta of type-2 diabetic animal models [21,24,25,26]. The functional impairment was supported by Western blotting data, which showed a reduction in Akt and eNOS proteins expression in the aorta of diabetic rats. A study by Georgescu et al. in 2011 [27] indicates that type-2 diabetes in combination with obesity causes much more severe changes, reducing the expression of Akt and eNOS, and inhibiting NO release within the arteriolar lumen in association with acetylcholine-mediated endothelium-dependent relaxation. However, Akther et al. in 2021 [28] and Zhong et al. in 2012 [29] have reported enhanced acetylcholine-mediated relaxation responses in aortic rings of male UC Davis type-2 diabetes mellitus rats and Goto-Kakizaki male rats, respectively. The discrepancies may be due to several factors, such as genetically modified rats, diet modification, and variation in the ages of rats used.

Endothelial dysfunction, diabetes, and cardiovascular diseases have all been associated with prolonged ER stress activation [30]. Prolonged ER stress has been demonstrated by the activation of UPR signal transducers, notably IRE-1 and ATF-6, in endothelial cells isolated from atherosclerosis-prone arteries [31]. Also, an ER pharmacological stressor known as tunicamycin was employed to induce ER stress in the coronary artery of endothelial cells, which resulted in endothelial dysfunction [32]. Tunicamycin causes oxidative stress by upregulating the mRNA expression of NADPH oxidase, the primary generator of ROS in the endothelium, which compromises endothelial function by lowering NO bioavailability [32]. Furthermore, the activity of eNOS and its expression in coronary endothelial cells were reduced after ER stress was induced [15]. The present study showed that inhibition of ER stress with TUDCA in diabetic rats improved acetylcholine-mediated relaxation compared to diabetic rats (Figure 7). The present observations are consistent with other studies demonstrating inhibition of ER stress with TUDCA-improved endothelial function in coronary arteries of SHR and diabetic animals [21,33,34].

In the aorta of control rats, all the pharmacological inhibitors have successfully inhibited acetylcholine-induced relaxation to varying degrees, showing that the relaxation of the aorta in control rats involved the activation of eNOS/PI3K/Akt, NAD(P)H oxidase, and the K_ATP_ channel. NO has been considered a major contributor to endothelium-dependent relaxation in large conduit arteries. The enzyme responsible for NO generation from the endothelium is eNOS. The Akt/PI3K pathway regulates the phosphorylation of eNOS at Ser1177, which results in increased synthesis of NO by this enzyme, and an enhanced vasodilator response. Furthermore, defective Akt/PI3K-dependent phosphorylation of eNOS contributes to the impairment of NO-mediated vasodilatation in several pathological conditions [25,35,36]. Many previous studies on acetylcholine-induced vasorelaxation supported this finding [37,38]. However, Kobayashi and Kamata in 2001 [39] did not support the idea that acetylcholine-induced endothelium impairment is due to the downregulation of eNOS and NO generation, but rather due to aberrant oxidative metabolism of NO.

The endothelial NAD(P)H oxidase generates ROS such as hydrogen peroxide (H_2_O_2_) and superoxide anion (O^2.^) as downstream metabolites [5]. Endogenous H_2_O_2_ may operate as an endothelium-derived vasodilator, causing vasodilation in various murine and human arteries [40,41]. In the present study, acetylcholine-mediated relaxation involved the activation of NAD(P)H oxidase, which might generate H_2_O_2_ that contributed at least in part to the vasorelaxation. Similarly, a previous study supported this result by demonstrating that NAD(P)H oxidase-induced ROS are involved in endothelium-dependent vasodilation in unaltered coronary blood arteries [42]. Reduced activation of NAD(P)H oxidase results in a decreased eNOS/Akt/PI3K signalling pathway, thereby leading to reduced NO bioavailability [42]. A variety of mechanisms cause H_2_O_2_-induced vasodilation, including changes in eNOS activation/expression, or protein kinase G, or the initiation of soluble guanylate cyclase, which are NO downstream targets for vasodilation [43]. Gao et al. in 2003 [44] and Fujimoto et al. in 2001 [45] have demonstrated that H_2_O_2_ can induce and activate eNOS. Also, the vasodilatory effects of H_2_O_2_ in the vasculature can be achieved via hyperpolarization mechanisms [46].

K_ATP_ channels are found throughout the body, but are abundant in the heart and smooth muscles [47]. They play a role in various physiological functions like regulating vascular tone and blood flow [7]. K_ATP_ channels consist of four regulatory sulphonylurea receptor subunits [8]. The activation of the endothelial K_ATP_ channel enhances the entry of Ca^2+^ into endothelial cells [10]. The increase in endothelial cell Ca^2+^ leads to the formation of CaM [11]. CaM may, at least in part, contribute to the activation of eNOS. Hutcheson and Griffith in 1994 [48] have shown that the endothelial K_ATP_ channel contributes to the release of NO in the aorta of rabbits. However, the entry of Ca^2+^ into the endothelial cells can also activate hyperpolarization mechanisms [10]. G-protein, protein kinase A and downstream stimulation of adenylate cyclase can activate the endothelial K_ATP_ channel. A study by Aziz et al. in 2017 [10] has supported the notion that the endothelial K_ATP_ channel plays an important role in endothelial-dependent vasorelaxation, which is consistent with this present study.

In the aorta of diabetic rats, maximal relaxations were almost abolished in the presence of LNAME, wortmannin, and apocynin, but not gliclazide. This showed that the eNOS/Akt/PI3K and NAD(P)H oxidase pathways play essential roles in acetylcholine-induced endothelium-dependent relaxation in the aorta of diabetic rats, while the endothelial K_ATP_ channel pathway contributes little to vasorelaxation in diabetes. Reduced acetylcholine-mediated relaxation through the K_ATP_ channel pathway in the diabetic rat model might be due to the reduced expression of the K_ATP_ channel. It has been reported that K_ATP_ channel expression was reduced in diabetic rats compared with non-diabetic rats [49]. Similarly, reduction in cardiac K_ATP_ channel expression is among the mechanisms that leads to heart dysfunction in the initial stages of diabetes [49]. In contrast to the present finding, Li et al. in 2020 [50] have shown that the endothelial K_ATP_ channel contributes more to relaxation in pathological conditions than physiological conditions. The differences could be due to animal variation and experimental protocols.

The present study demonstrated that maximal relaxation of the aorta of diabetic rats receiving TUDCA was almost abolished in the presence of all pharmacological inhibitors. This finding suggested that inhibition of ER stress in diabetes profoundly restores acetylcholine-induced endothelium-dependent relaxation by reverting the signalling pathways similar to that of control rats. Similarly, studies by Choi et al. in 2016 [21] and Choi et al. in 2016 [22] have demonstrated that TUDCA was able to reduce ER stress and improve endothelium-dependent relaxation. Wang et al. in 2015 [51] showed that TUDCA or 4-phenylbutyric acid (PBA-4) ameliorate ER stress that mediates homocysteine-induced endothelial dysfunction. Furthermore, ER stress plays a significant role in the pathophysiology of cardiometabolic disorders and TUDCA may be a possible treatment for both fatty liver disease and the diabetic vascular problems that accompany it [52]. In fact, recent findings by Choy et al. in 2017 [33] supported the notion that co-administration of TUDCA with paeonol or tempol improved NO bioavailability and endothelium-dependent relaxation. Spitler et al. in 2013 [53] and Kassan et al. in 2012 [19] have demonstrated that acetylcholine-induced vascular relaxation was greatly improved with TUDCA administration. This data also indicated that ER stress contributes, at least in part, to the impairment of acetylcholine-mediated endothelium-dependent relaxation via the K_ATP_ channel, which is in line with the study by Matsumoto et al. [54]. Similarly, previous studies by Matsumoto et al. in 2016a [54] and Matsumoto et al. in 2016b [55] have demonstrated that prolonged activation of ER stress can lead to the impairment of the K_ATP_ channel [54], which was restored following treatment with TUDCA [55]. However, Vettorazzi et al. in 2016 [56] have shown that TUDCA had no influence on K_ATP_ channel activity and intracellular Ca^2+^ signals, suggesting that such mechanisms are not involved. TUDCA was administered systemically for two weeks in the present study, while Vettorazzi et al. in 2016 [56] pre-incubated the aortic tissues with TUDCA for 30 min during the functional study.

ER stress is linked to oxidative stress in a vicious spiral, contributing to endothelial dysfunction [15]. Oxidative stress involves overproducing ROS, which exceeds the antioxidant ability to scavenge them. The oxidative condition of a cell has a direct impact on ER homeostasis. Many diseases, such as diabetes and obesity, are etiologically linked to ROS. Similarly, most defense mechanisms, like glutathione peroxidases, catalase, and SOD against excessive ROS production, tend to be compromised in disease conditions. SOD also competes with NO for O^2−^ scavenging, inhibiting NO from attaching to O^2−^ and increasing NO availability [57]. In this present study, the aorta of diabetic rats showed higher SOD activity than control rats. This result was comparable to previous studies [58,59,60,61,62]. This increase in SOD activity could be a possible coping response due to the excessive production of ROS that occurs with diabetes. However, a few researchers also reported on lower SOD activity in diabetes [24,63,64,65,66]. Chou & Tseng, in 2017, [67] demonstrated that there were no differences in SOD activity among the three groups in type-2 diabetes patients with diabetic nephropathy. In the present study, the SOD activity in diabetic rats receiving TUDCA was observed to be decreased; however, it was not a complete recovery as they were still higher when compared with the control rats. This finding suggested that TUDCA can alleviate the effect of ER stress due to the excessive production of ROS via its antioxidant action. However, an overabundance of ROS might deplete the body’s antioxidant capability. In support of our hypothesis, Wang et al. in 2012 [68] have shown that using TUDCA in rats with liver fibrosis did not fully restore their SOD activity. However, contrary to our findings, a few studies have shown that the use of TUDCA can elevate SOD activity in ER stress neonatal rat cardiomyocytes [69], retinal degeneration [70] and spinal cord injury in mice [71].

Another critical biomarker in measuring oxidative stress is MDA, a byproduct of lipid peroxidation from free radicals that are hazardous to cells [72]. MDA refers to the extent of cellular peroxidative damage caused by elevated ROS levels. The existence of vascular oxidative damage was confirmed in this present study by elevated MDA levels in the aortic tissue of diabetic rats. Similarly, elevated MDA levels were reported in the aortic tissues of STZ-induced diabetic rats [73,74] and fructose-fed STZ-induced diabetic rats [75]. This is due to the accumulation of misfolded or unfolded proteins that activate a process called ER stress. Prior to activation of ER stress, the cells tend to trigger a process called the ER stress response or UPR. The essence of this process is to re-establish the ER lumen homeostasis. The most common findings on MDA in type-2 diabetes with or without complications were significant elevations of MDA when matched with controls [66] in both animals and humans. In this study, MDA levels were not reduced in diabetic rats after inhibition of ER stress with TUDCA compared with DM. Therefore, TUDCA did not produce complete recovery of MDA levels as a measure of oxidative stress in accordance with previous studies [76,77]. Similarly, Mantopoulos et al. in 2011 [78] demonstrated that TUDCA is ineffective in inhibiting necrotic and apoptotic pathways induced by retinal detachment. Lipid peroxidation plays a vital role in developing many diseases and apoptosis. However, contrary to our findings, a study by Alhasani et al. in 2020 [70] has shown that TUDCA on retinal pigment epithelium can significantly reduce MDA levels.

The stress response triggered by metabolic perturbations includes cellular inflammatory responses activated by ER stress. These cellular inflammatory responses can aid in attenuating or progressing ER stress. This process solely depends on the intensity of ER stress or the UPR response’s ability to re-establish ER homeostasis. Activation of the UPR has been linked to the production of many pro-inflammatory cytokines, like IL-6 and TNF-α [79]. In the present study, the cellular inflammatory cytokine IL-6 was elevated in the aorta of diabetic rats when matched with control rats. This result is an indication of increased vascular inflammation. The present finding was supported by previous studies on diabetic rats induced by STZ [75,80]. Besides, a study by Choy et al. in 2021 [81] had shown increased IL-6 levels in db/db mice. However, treatment with TUDCA in diabetic rats did not affect the level of IL-6, and it was comparable to that of diabetic rats without treatment. This is consistent with the study that shows TUDCA did not affect the levels of IL-6 in isolated biliary epithelial cells [82]. In addition, Ocana et al. in 2016 [83] demonstrated that TUDCA treatment, as an inhibitor of ER stress, did not completely prevent all changes in beta cells caused by pro-inflammatory cytokines.

## 4. Materials and Methods

### 4.1. Animals

The experimental protocol was approved by the Universiti Sains Malaysia Institutional Animal Care and Use Committee [USM/IACUC/2019/(116)(965)]. Male Sprague–Dawley rats aged 8–10 weeks and weighing 250–300 g were used in the experiment. The rats were housed in cages throughout the experiment with a 12-h light/dark cycle and a temperature of 25 ± 2 °C. During the first week of the acclimatization period, all rats were allowed access to standard commercial pellets (Gold Coin Feedmils, Port Klang, Malaysia) and water ad libitum. Following the acclimatization phase, rats were divided into two groups: control (CON, n = 9) and diabetic (n = 18). Rats in the CON groups were fed standard commercial pellets, while diabetic rats were given modified high-fat diet pellets to develop obesity (Azemi et al., 2020). After four weeks on their respective diets, rats in the diabetic group were intraperitoneally (i.p.) injected with a single dose of streptozotocin (STZ, 40 mg/kg, dissolved in 0.1 M sodium citrate buffer, pH 4.5). The rats in the CON group were given an equivalent amount of sodium citrate buffer (1 mL/kg, i.p) [26,84,85]. Blood samples were taken from the tail tip one week after STZ injection and used to assess fasting blood glucose (FBG) with a glucometer (Accu-check, Roche Diagnostic, Indianapolis, IN, USA). Diabetic rats were defined as having an FBG of more than 11.1 mmol/L (Perry et al., 2001). Diabetic rats were divided into two groups at random: (I) diabetic rats (DM, n = 9) and (II) diabetic rats receiving TUDCA (150 mg/kg/day i.p. for the last two weeks of the experiment) (DMT, n = 9). All rats were sacrificed using a mixture of ketamine (300 mg/kg, i.p.) and xylazine (30 mg/kg, i.p.) at the end of week 15.

### 4.2. Measurement of Blood Pressure

The non-invasive tail-cuff approach was used to measure BP in a warmed, conscious, and restrained state utilizing BP monitoring equipment (Mouse and Rat Tail Cuff Method Blood Pressure Systems, IITC Life Science, Victory Blvd Woodland Hill, USA). Before actual recordings were acquired, rats were trained for one week on the device. BP measurements were taken every week for the first four weeks, then at two-week intervals until the end of the 15th week. For each rat, six readings were taken at each time point, and the average was documented.

### 4.3. Functional Study

The rat’s thoracic aorta was removed and promptly immersed in an ice-cold, oxygenated physiological saline solution [118 mM sodium chloride (NaCl), 4.7 mM potassium chloride (KCl), 2.0 mM calcium chloride dihydrate (CaCl_2_•2H_2_O), 1.18 mM (MgSO_4_•7H_2_O), 25 mM sodium hydrogen carbonate (NaHCO_3_), 1.2 mM potassium dihydrogen phosphate (KH_2_PO_4_), and 5.5 mM D-glucose]. The thoracic aorta was cleaned of fat and connective tissue before being cut into 3 mm rings. The rings were suspended with two stainless steel hooks in organ bath chambers filled with 20 mL of physiological saline solution. The bathing solution was kept at 37 °C and constantly bubbled with carbogen (95% O_2_ and 5% CO_2_) at a pH of 7.4. All rings were stretched to a resting tension of 1.0 g and equilibrated for 60 min. Subsequently, the rings were contracted with KCl (60 mM) to obtain a reference contraction and confirm smooth muscle viability. After washing, the rings were precontracted with phenylephrine (1 × 10^−6^ M) to achieve a steady contraction, and then acetylcholine (1 × 10^−6^ M) was added to evaluate for the presence or absence of functional endothelium. To study endothelium-dependent relaxation, aortic rings were precontracted with phenylephrine (1 × 10^−6^ M) and subjected to cumulative doses of acetylcholine (1 × 10^−9^–1 × 10^−5^ M). In some preparations, the rings were incubated with pharmacological inhibitors for 30 min before being subjected to phenylephrine and acetylcholine. The pharmacological inhibitors were: L-Nitro-Arginine Methyl Ester (L-NAME) (1 × 10^−4^ M) (inhibitor of eNOS), apocynin (1 × 10^−4^ M) (inhibitor of nicotinamide adenine dinucleotide phosphate oxidase (NAD(P)H oxidase), wortmannin (1 × 10^−7^ M) (inhibitor of phosphatidylinositol-3-kinase (PI3K)), and gliclazide (1 × 10^−4^ M) (inhibitor of K_ATP_ channel)) [25,26,86,87,88,89].

### 4.4. Western Blotting

The thoracic aorta was homogenized in lysis Radioimmunoprecipitation assay buffer (RIPA buffer) (Sigma Chemical Co., St. Louis, MO, USA) containing a 0.05% protease inhibitor cocktail (Sigma Chemical Co., St. Louis, MO, USA). The supernatants were collected after centrifugation at 3000× *g* for 20 min at 4 °C. The protein concentration of the supernatant was determined using a protein determination kit (Cayman Chemicals, Ann Arbor, MI, USA). Protein homogenates (30 µg) were subjected to sodium dodecyl sulphate polyacrylamide gel electrophoresis. After that, the proteins were transferred to polyvinylidene difluoride membranes (Millipore Corp., Billerica, MA, USA). The membranes were then probed with primary antibodies against eNOS (1:1000; Cell Signaling, Danvers, MA, USA), Akt (1:1000; Cell Signaling, Danvers, MA, USA), BiP (1:1000; Cell Signaling, Danvers, MA, USA), PERK (1:1000; Cell Signaling, Danvers, MA, USA), and β-actin (1:1000; Cell Signaling, Danvers, MA, USA) at 4 °C for 16 h. After washing, the membranes were probed with horseradish peroxidase-conjugated polyclonal secondary antibody (1:1000; Cell Signaling, Danvers, MA, USA) for one hour at room temperature. The protein bands were detected using Signal Fire ECL reagent (Cell Signaling, Danvers, MA, USA) and viewed with FluorChem M (Santa Clara, CA, USA). The intensity of the protein bands were quantified with ImageJ software. The relative presence of eNOS, Akt, BiP, and PERK were calculated based on the ratio of the intensity of all the protein bands to the corresponding β-actin.

### 4.5. Biochemical Analysis of Thoracic Aorta Tissue Lysate

The supernatants of thoracic aorta tissue lysate were prepared as described in the Western blotting method. Levels of oxidative stress markers [superoxide dismutase (SOD) and malondialdehyde (MDA)] and inflammatory markers [tumour necrosis factor-Alpha (TNF-α) and interleukin 6 (IL-6)] were measured using commercial enzyme-linked immunosorbent assay (ELISA) kits according to the manufacturer’s instructions. The (QAYEE-BIO) ELISA kits for SOD, MDA, TNF-α, and IL-6 were purchased from Qayee biotechnology (Shanghai, China). The SOD activity was expressed as units per milligram of protein [90]. The MDA level was expressed as nanomoles per milligram of protein. The levels of TNF-α and IL-6 were expressed as picograms per milliliter [91].

### 4.6. Statistical Analysis

All data were analysed using GraphPad Prism version 9.0 (San Diego, CA, USA). Relaxation was expressed as a percentage of the tension generated by 1 × 10^−6^ M of phenylephrine. Analysis of variance (ANOVA) was used to examine between-group differences, followed by Tukey’s post-hoc tests. Data were presented as mean ± SEM. A *p*-value less than 0.05 was considered statistically significant (*p* < 0.05).

## 5. Conclusions

Acetylcholine-induced endothelium-dependent relaxation was impaired in the aorta of diabetic rats compared to control rats. The inhibition of ER stress with TUDCA in diabetic rats improved acetylcholine-mediated relaxation. This was associated with decreased ER stress markers, BiP and PERK, and increased eNOS and Akt protein expressions. ER stress inhibition in diabetic rats also improved acetylcholine-mediated relaxation through the eNOS/PI3K/Akt pathway, NAD(P)H oxidase, and endothelial K_ATP_ channel. ER stress contributes, at least in part, to the pathogenesis of endothelial dysfunction in association with type-2 diabetes. The use of TUDCA attenuates the effect of ER stress on the endothelium. Therefore, targeting ER stress in type-2 diabetes cannot be overemphasized.

## Figures and Tables

**Table 1:** Percentage of maximal relaxations to acetylcholine in the rat aorta.

Groups	CON	DM	DMT
	Maximal relaxation, %		
W/O inhibitor	89.89 ± 1.63 ^a^	42.57 ± 1.68	84.91 ± 3.44 ^a^
LNAME	5.71 ± 1.91 ^b^	11.56 ± 2.34 ^b^	16.84 ± 2.17 ^b^
Wortmannin	14.76 ± 2.80 ^b^	20.13 ± 2.45 ^b^	19.13 ± 1.95 ^b^
Apocynin	13.71 ± 2.95 ^b^	13.24 ± 2.86 ^b^	20.23 ± 3.34 ^b^
Gliclazide	21.43 ± 4.86 ^b^	32.56 ± 4.30	25.71 ± 2.03 ^b^

Abbreviation: W/O: without inhibitor, CON; Control, DM; Diabetes, DMT; Diabetes receiving TUDCA and LNAME; L-Nitro-Arginine Methyl Ester. ^a^ *p* < 0.05, vs. DM, ^b^ *p* < 0.05, vs. W/O inhibitor.

## Data Availability

Not applicable.
